# The impact of emotional context on neural substrates of respiratory sensory gating

**DOI:** 10.3389/fnins.2022.1004271

**Published:** 2022-10-20

**Authors:** Pei-Ying S. Chan, Wen-Pin Chang, Chia-Hsiung Cheng, Chia-Yih Liu, Andreas von Leupoldt, Ai-Ling Hsu, Changwei W. Wu

**Affiliations:** ^1^Department of Occupational Therapy, College of Medicine, and Healthy Ageing Research Center, Chang Gung University, Taoyuan, Taiwan; ^2^Department of Psychiatry, Chang Gung Memorial Hospital at Linkou, Taoyuan, Taiwan; ^3^Department of Occupational Therapy, Rocky Mountain University of Health Professions, Provo, UT, United States; ^4^Laboratory of Brain Imaging and Neural Dynamics (BIND Lab), Chang Gung University, Taoyuan, Taiwan; ^5^Research Group Health Psychology, University of Leuven, Leuven, Belgium; ^6^Bachelor Program in Artificial Intelligence, Chang Gung University, Taoyuan, Taiwan; ^7^Graduate Institute of Mind, Brain and Consciousness, Taipei Medical University, Taipei, Taiwan; ^8^Brain and Consciousness Research Center, Shuang-Ho Hospital, Taipei Medical University, Taipei, Taiwan

**Keywords:** respiratory sensations, functional magnetic resonance imaging, human respiratory sensory gating, positive emotional context, cortical neural substrates

## Abstract

Psychological challenges have been found to impact respiratory symptom perception in healthy individuals as well as in patients with various neurological disorders. Human respiratory sensory gating is an objective measure to examine respiratory sensory information processing of repetitive respiratory mechanical stimuli in the central nervous system. With this electrophysiological method, patients with higher anxiety levels showed reduced respiratory sensory gating function in the cortex, and increased symptom perception. In addition, positive emotional contexts were found to increase the respiratory sensory gating function using RREPs. However, neural substrates related to emotional impacts on respiratory sensory gating remain still unclear. In the present study, we examined the emotion processing of respiratory sensory gating using functional magnetic resonance imaging. We hypothesized that positive compared with neutral stimuli would result in reduced brain activations in cortical areas with the paired occlusion paradigm. Thirty-five healthy adults participated in this event-designed fMRI experiment. Paired inspiratory occlusions (two transient occlusions with a 500 ms inter-stimulus-interval are delivered during one inspiration) were provided using an external trigger outside of the scanner. At least 40 paired inspiratory occlusions were collected for each trial. The experiment contained three runs during which participants underwent 12 min for the paired inspiratory occlusion paradigm while watching a fixation cross (the control condition), neutral and positive emotional picture series. The order of emotional picture series was randomized across the participants. Our results revealed an overall trend of reduction of brain activity from the neutral (minus fixation) condition, to the pleasant (minus fixation) condition. For bilateral thalamus and primary visual cortices, there was no significant difference in neural activation between the two contrasts of pleasant (Contrast_P–F_) and neutral condition (Contrast_N–F_). The activation of the mid-cingulate and the orbitofrontal cortex was lower in Contrast_P–F_ compared to Contrast_N–F_. In conclusion, our results suggest that emotional context, especially positive valence, modulates neural correlates in middle cingulate cortex and orbitofrontal cortex in terms of respiratory sensory gating. Future studies are recommended to test emotional impacts on respiratory sensations in patients with neurological disorders.

## Introduction

Previous research has shown that emotions affect mental and physical health and wellbeing (DeSteno et al., 2013). Unhealthy or negative emotions can deplete vital energy and increase the propensity for chronic diseases (Locke et al., 2004; Roest et al., 2010; Hartono et al., 2012). Specifically, the adverse impact of psychological distress on disease exacerbation, mortality, and lung function is well documented (Felker et al., 2001; McCathie et al., 2002; Yohannes et al., 2005; Ng et al., 2007). For example, patients with chronic diseases such as chronic obstructive pulmonary disease (COPD) patients with significant impairments of lung function often suffer from negative emotions including feelings of worry, fear, and sadness (Strang et al., 2014) as well as elevated levels of depression and anxiety (Yohannes and Alexopoulos, 2014). Similar findings have been observed in patients with neurological disorders such as Parkinson’s disease (Baille et al., 2019a,b; Das et al., 2019). Moreover, previous psychophysiological research has demonstrated increased respiratory sensations in patients with COPD during experimentally induced short-lasting negative affective states (von Leupoldt et al., 2010c) or in those patients with higher trait-like long-term forms of negative affectivity (Livermore et al., 2008; von Leupoldt et al., 2011; von Leupoldt and Denutte, 2020). Similarly, patients with Parkinson’s disease with high levels of depression and anxiety showed stronger symptoms of breathlessness (Baille et al., 2019a). In addition, Masaoka and Homma (2001) revealed that unpleasant emotions changed breathing patterns, and especially respiratory rate was positively correlated with individual state and trait anxiety levels (Masaoka and Homma, 2001). Leander et al. (2014) also found a connection between respiratory symptoms and psychological status and suggested that improving psychological status may be a valuable method to decrease respiratory symptoms in patients suffering from pulmonary diseases, such as COPD (Leander et al., 2014).

Psychophysiological studies further confirmed the relationship between emotions and the neural processing of respiratory sensations (Bogaerts et al., 2005; Chan et al., 2014, 2016), which is significant for human survival and can be measured by the respiratory-related evoked potential (RREP) in the electroencephalogram (Chan et al., 2015a; Herzog et al., 2021). With the single-obstruction (odd-ball) paradigm, von Leupoldt et al. (2010a) found that the amplitude of the RREP P3 component decreased in pleasant and unpleasant emotional contexts compared to a neutral emotional context, indicating that the processing of respiratory sensory input was modulated by emotions (von Leupoldt et al., 2010a). Two recent studies confirmed the observation that experimentally induced negative emotional states modulate the perception of respiratory sensations and their neural processing as evidenced by significant amplitude modulations of RREP components P2 and P3 (Herzog et al., 2019a,b). Another RREP study showed that the P3 amplitude of a high trait-like anxious group was smaller in a neutral emotional context compared to an unpleasant emotional context (von Leupoldt et al., 2011). In contrast, the P3 amplitude of a low anxious group was higher in the neutral emotional context compared to the unpleasant emotional context, which the authors interpreted such that respiratory sensations in participants with higher anxiety levels might demand more attentional and neural resources in an unpleasant affective context (von Leupoldt et al., 2011). Also in patients with COPD, a recent study showed increased RREP amplitudes compared to healthy controls, which were related to increased perception of respiratory sensations, presumably due to the higher relevance and attentional as well as neural demand in the patients (Reijnders et al., 2020).

The paired-obstruction RREP paradigm (i.e., obstructing twice within a single inspiration), another RREP method, is used to quantify a neural filter mechanism for respiratory sensations called “respiratory sensory gating,” i.e., the ability of the brain to suppress the processing of repetitive respiratory sensory stimuli (Chan and Davenport, 2008; von Leupoldt et al., 2011). This paradigm employs paired identical inspiratory obstructions of 150 milliseconds (ms) each (first stimulus/S1 and second stimulus/S2) with an inter-stimulus-interval (ISI) of 500 ms, resulting in a RREP N1 peak amplitude of S2/S1 ratio, which is an index of the amount of neural information filtered “in” vs. filtered “out” (Chan and Davenport, 2010a). Previous studies have shown that stress and anxiety are related to an altered respiratory sensory gating function (Chan and Davenport, 2010b; Chan et al., 2012, 2014, 2015b; Herzog et al., 2018). For instance, Chan et al. (2012) found that the neural throughput of the second stimulus may have been disinhibited at the subcortical level in anxious individuals, causing over-perception (Chan et al., 2012). Chan et al. (2015b) further showed that individuals with generalized anxiety disorder exhibited a decreased respiratory sensory gating due to a smaller response to the novel stimulus than the healthy controls (Chan et al., 2015b). Moreover, Chenivesse et al. (2014) demonstrated that short-lasting negative emotional states as induced by affective picture viewing reduced the respiratory sensory gating function when compared to a neutral emotional state (Chenivesse et al., 2014). Besides the effect of negative affect and anxiety on respiratory sensory gating, positive emotional contexts also gained attention in previous research (Tsai et al., 2013; Chan et al., 2016). For example, Chan et al. (2016) showed that a positive emotional context, relative to a neutral emotional context, was related to a better respiratory sensory gating function as evidenced by a reduced N1 peak S2/S1 ratio (Chan et al., 2016).

However, although emotional modulations of the RREP were previously demonstrated (von Leupoldt et al., 2013), studying the precise brain areas involved in the cortical and subcortical processing of respiratory sensations as reflected by the RREP method remains challenging and lacks precision. Past source analyses and animal studies suggested that the somatosensory cortex, somatosensory association cortex (SII), prefrontal, premotor, and supplementary motor cortices, as well as parietal areas, thalamus, and brainstem, were activated during short respiratory occlusions (Yates et al., 1994; Davenport et al., 1996; Logie et al., 1998; von Leupoldt et al., 2010b). More recent studies have used functional magnetic resonance imaging (fMRI) to compensate for the poor spatial resolution of the RREP method. These fMRI studies revealed activation patterns of neural substrates of respiratory sensations elicited by short single inspiratory occlusions, including cortical (sensorimotor cortices, frontal lobes, supramarginal gyrus, cingulate cortex, insular cortex) and subcortical (thalamus, hippocampus, and caudate) areas (Jack et al., 2010; Chan et al., 2018, 2019, 2022b). In addition, more prolonged respiratory obstructions demonstrated additional neural activation of the amygdala, cingulate cortex, and insular cortex under conditions of resistive loaded breathing-induced dyspnea (Peiffer et al., 2001; von Leupoldt et al., 2008, 2009a, 2010b; Faull et al., 2017; Vlemincx et al., 2021), which were also areas associated with elevated levels of negative affect such as anxiety or increased unpleasantness of breathlessness (von Leupoldt et al., 2008, 2009a,b, 2010b). In addition, Chan et al. (2019) found an association between anxiety levels and activation of the precuneus and inferior parietal gyrus in the right hemisphere in response to short inspiratory occlusions (Chan et al., 2019).

However, the specific cortical activation patterns underlying the modulation of respiratory sensory gating during positive emotional contexts remain unclear. Therefore, the present fMRI study aimed to determine the neural substrates of paired inspiratory occlusions while viewing positive emotional pictures in healthy volunteers. Specifically, we expected to observe different levels of neural activation in emotion-related brain areas, including the cingulate cortex and insula as well as further cortical regions, such as sensorimotor cortices, frontal lobes, and supramarginal gyrus.

## Materials and methods

### Study participants

Thirty-five healthy adults of at least 20 years of age participated in the study. All participants self-reported to have no respiratory, cardiovascular, neurological, or psychiatric diseases. In order to comply with the fMRI room requirements, those with metal implants including denture/braces or with a pacemaker were excluded from the study. In addition, potential participants with a head circumference over 60 cm and/or with claustrophobia were also not complying with the current fMRI setup and were therefore excluded. All study procedures were approved by the Institutional Review Board of the Chang Gung Medical Foundation.

### Experiment protocol

The participants were required to perform a spirometry pulmonary function test (Cardinal Health Inc., Dublin, OH, USA) based on the American Thoracic Society and European Respiratory Society guidelines (Miller et al., 2005). All participants passed the minimum requirement of the Forced Expiratory Volume in 1 s (FEV1) of at least 70% of predicted normative values to be eligible to participate in the study. Upon completion, the participants were instructed to lie supine in the fMRI scanner. During the experiment, the participants were instructed to breathe through the facemask as normally as possible regardless of the occasional obstructed breaths.

For the details of respiratory apparatus, please refer to a previous study of Chan et al. (2019). Briefly, the inspiratory port of the two-way non-rebreathing valve at the facemask was connected *via* tubing to a customized occlusion valve placed approximately 3 m away from the fMRI scanner (Hans Rudolph Inc., Kansas City, USA). The occlusion valve was connected to a solenoid of a customized trigger and a pressure tank outside of the scanner. Occasional inspiratory occlusions were elicited by the closing of the occlusion valve (triggered manually by the experimenter). Two 150-ms inspiratory occlusions with a 500-ms inter-stimulus-interval were provided during the inspiratory phase every 2–4 breaths. At least 40 successful paired inspiratory occlusions per condition were collected for data analysis. Every participant underwent three paired inspiratory occlusion conditions, each lasting approximately 12 min. The first condition was always the control condition. For this control condition, the participant was instructed to focus on the fixation on the screen. The control condition was followed by the neutral and positive emotional context conditions with the sequence of these emotional conditions being randomized across participants. For the emotional context conditions, participants were instructed to watch a series of neutral emotional pictures during the neutral condition and a series of pleasant emotional pictures during the positive condition.

The emotional pictures used to create the positive and neutral emotional context were selected from the International Affective Picture Series (Lang et al., 2008). The IAPS is a standardized and validated instrument used in various studies to induce different short-lasting emotional states in study participants (Lang et al., 2008). The positive and neutral conditions were composed of 120 pictures selected from each category. Every picture was presented on the screen for 6 s. The participants viewed the picture series via a mirror mounted on the head coil. After each scan, the participants rated the perceived level of breathlessness using a visual analog scale (VAS), ranging from 0 to 100 (0 = no breathlessness and 100 = maximal level of breathlessness), as well as the subjective level of valence and arousal using the Self-Assessment Manikin (SAM) scale, ranging from 1 to 9 (1 = no arousal or not pleasant at all, and 9 = maximal arousal or pleasantness).

### Image acquisition

The fMRI study was performed using a 3-T Magnetom scanner (Siemens MAGNETOM Prisma, Erlangen, Germany) in the National Taiwan University, Taipei, Taiwan. During the scan, the participants laid supine and their heads were comfortably positioned inside a standard head coil, which was padded with sponges for head immobilization. The participants were wearing earplugs in order to minimize interferences with the scanner noise. During the fMRI experiments, thirty-two continuous axial slices (3-mm thickness) were acquired by using a gradient echo, echo-planar imaging sequence to acquire blood oxygen level-dependent (BOLD) data (TR = 2000 ms, TE = 30 ms; flip angle = 90 degrees; matrix = 64 × 64; field of view = 220 × 220 mm). The total acquisition time was 12 min for each condition (360 scans).

### Data analysis

The fMRI data was pre-processed using SPM8 (Welcome Department of Cognitive Neurology, Institute of Neurology, London, UK) on the MATLAB platform (The MathWorks, Inc., Natick, MA, USA) and AFNI (Cox, 1996). All images were motion corrected and realigned to the first imaging volume, spatially normalized into standard Montreal Neurologic Institute space and smoothed with an isotropic Gaussian kernel of 6-mm full-width at half-maximum.

At the first-level, the statistical analysis was performed with the general linear model (GLM) across the three experimental conditions: The fixation condition, the neutral emotional context, and the positive emotional context. A high-pass filter with a cut-off frequency of 128 s per wave was used to eliminate baseline drifts. Specifically, 12 regressors were considered in the GLM: one for onset timing of the corresponding respiratory occlusion condition convolved with the canonical hemodynamic response function, two for time and dispersion derivatives of the first regressor, one for baseline intensity, six for motion parameters, one for the voxel time series with highest temporal standard deviation, and the last one for the averaged time series within a cerebrospinal fluid (CSF) mask. The last two regressors were used as nuisance regressors to minimize the impact of physiological noise caused by baseline respiration (Birn et al., 2006). The CSF mask was generated by the labeled bilateral lateral ventricles provided by Neuromorphometrics, Inc.,^1^ with single-voxel erosion. After model estimation, the linear contrasts of interest were generated on the basis of the ensuing parameter estimates for each participant. The contrasts compared the averaged neuronal activity (BOLD response) across the baseline conditions (without occlusions) with the averaged neuronal activity for every condition to examine the main effect of respiratory sensation induced by the paired-occlusion paradigm in the fixation control, neutral, and positive contexts. Next, beta estimates were used in the second-level (group-level) analysis in SPM to identify the brain regions that were significantly activated in each condition and in the between-condition contrasts. The one-sample group activation maps were calculated separately for each condition with the multiple comparison correction of the family-wise error rate (FWE) corrected *p* < 0.05 and a cluster threshold of 20 voxels. Additionally, the contrast maps, i.e., the contrast between neutral context and fixation condition (Contrast_N–F_) as well as the contrast between positive context and fixation condition (Contrast_P–F_), were conducted using AFNI 3dClustSim correction method with autocorrelation function (corrected *p* < 0.05), where the parameter was set with uncorrected *p* < 0.001 and a cluster threshold of 65 voxels.

#### Region of interest analysis

In the region of interest (ROI) analysis, β-values were extracted and averaged over a whole ROI. One set of ROIs were defined based on the between-condition contrast maps (Contrast_N–F_ compared with Contrast_P–F_). In addition, ROIs from the automated anatomical labeling (AAL) templates were chosen on the basis of previous studies to test the effects of emotional contexts on paired-occlusion elicited brain areas, which included bilateral anterior cingulate cortex, insula, middle cingulate cortex, thalamus, precentral cortex, postcentral cortex, and calcarine fissure (Tzourio-Mazoyer et al., 2002; Chan et al., 2016, 2019, 2022b,a).

#### Statistical analysis

Descriptive statistics were performed for analyzing personal characteristics, including gender, age, and pulmonary function data. Self-reported ratings of valence and arousal were compared between the neutral condition and pleasant emotional condition, and levels of breathlessness were compared between the three conditions using a one-way repeated measures analysis of variance (RMANOVA) with R software (*ezANOVA* version 4.4.0). The Contrast_N–F_ was compared with theContrast_P–F_ using their β contrasts with the paired *t*-test or Wilcoxon signed-rank test, depending on the results of the normality test. The statistical significance level was set as *p* < 0.05.

## Results

A total of 35 participants (22 females; mean age = 24.11 ± 3.95 years) completed the experiments. Table 1 shows the participants’ baseline characteristics. The average FEV1 was 3.20 ± 0.57 L (82.44 ± 9.00% of predicted values), and the FEV1/FVC ratio was 107.42 ± 6.63%. The average levels of perceived breathlessness reported for the fixation, neutral, and positive condition were 27.63 ± 21.42 vs. 30.32 ± 23.66 vs. 29.66 ± 25.16, respectively (*p* > 0.1). The perceived level of valence was significantly lower in the neutral compared to the positive context (4.91 + 1.70 vs. 6.51 ± 1.42, *p* < 0.01), whereas the level of arousal was not different between the two emotional contexts (3.53 ± 1.85 vs. 2.74 ± 1.68, *p* > 0.1).

**TABLE 1:** Baseline characteristics and self-reported ratings.

Characteristics	M ± SD
N	35
Age (year)	24.11 ± 3.95
Gender (Male/Female)	13/22
Education level (high school/bachelor/master)	1/27/7
FEV_1_ (L)	3.20 ± 0.57
FEV_1_ of predicted value (%)	82.44 ± 9.00
FEV_1/_FVC (%)	107.42 ± 6.63
VAS ratings for breathlessness (control vs. neutral vs. positive context)	27.63 ± 21.42 vs. 30.32 ± 23.66 vs. 29.66 ± 25.16
SAM Valence in neutral vs. positive context	4.91 + 1.70 vs. 6.51 ± 1.42*
SAM arousal in neutral vs. positive context	3.53 ± 1.85 vs. 2.74 ± 1.68

FEV1, Forced expiratory volume in 1 s; FVC, Forced vital capacity; VAS, Visual Analogue Scale; SAM, Self-Assessment Manikin.

*p < 0.001.

**FIGURE 1:**
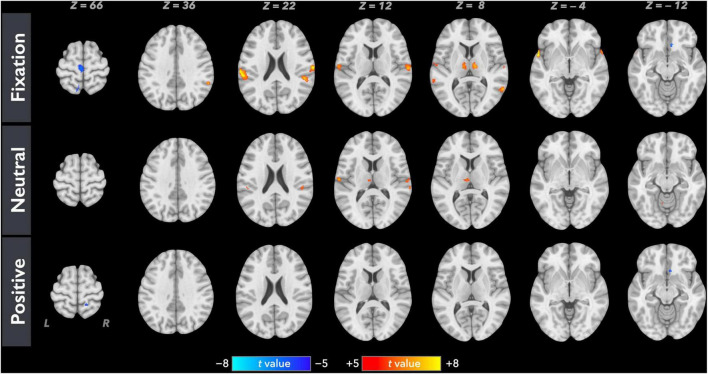
Averaged neural activation maps for the study participants (*N* = 35) in the fixation, neutral, and positive conditions (FWE-corrected, *p* < 0.05 and cluster size > 20 voxels).

Figure 1 shows the average brain activation maps for the study participants in the control and experimental conditions (FWE-corrected, *p* < 0.05 and cluster size > 20 voxels). Overall, paired inspiratory occlusions elicited various brain activities in various brain regions including bilateral thalamus, sensorimotor cortices, parietal cortices, cingulate cortices, and frontal gyrus. The level of activation was observed to be higher in the fixation (control) condition, followed by the neutral emotional condition, and the positive emotional condition. In addition, Contrast_N–F_ as well as Contrast_P–F_ were conducted using the AFNI 3dClustSim correction method with autocorrelation function. The brain areas above the 3dClustSim threshold are shown in Table 2. The two ROIs showing different activation levels between Contrast_N–F_ and Contrast_P–F_, the middle cingulate cortex (MCC) and the orbitofrontal cortex (OFC), were chosen from Table 2 for further ROI analysis.

**TABLE 2:** Brain areas showing significant differences in between-condition comparisons.

Brain area	Beta values (Mean ± SEM)		Cluster size	X	Y	Z
*C*_N–F_ > *C*_P–F_	Neutral	Positive				
Middle cingulate cortex	−0.03 ± 0.06	−0.27 ± 0.10	71	18	−18	42
Orbitofrontal cortex	−0.37 ± 0.14	−0.72 ± 0.14	67	6	38	−22

C_N–F_, Contrast between the neutral and fixation conditions; C_P–F_, Contrast between the pleasant and fixation conditions. Coordinates are shown in Montreal Neurological Institute (MNI) space.

**FIGURE 2:**
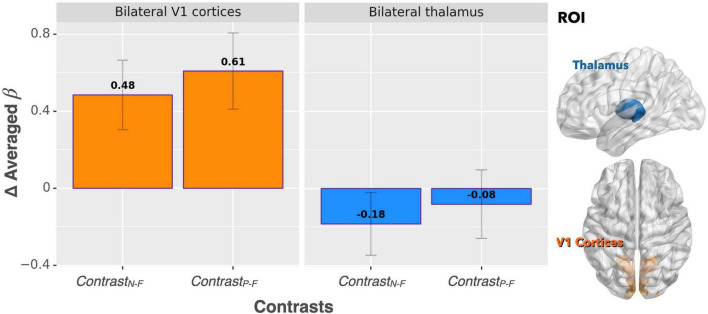
Beta value (averaged over the whole ROIs) comparisons between Contrast_N–F_ and Contrast_P–F_ for bilateral thalamus and V1 cortices. No significant difference was detected between the two contrasts in terms of activation in either ROI.

**FIGURE 3:**
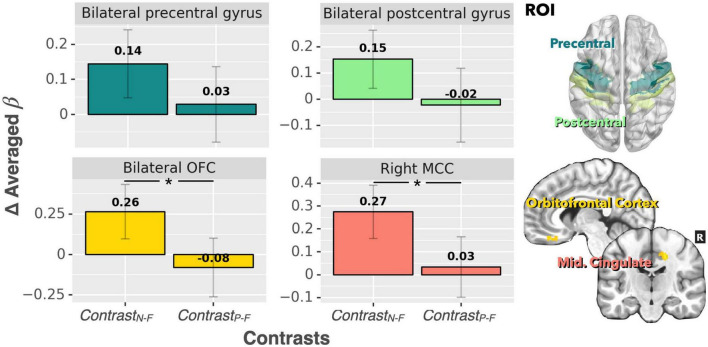
Beta value (averaged over the whole ROIs) comparisons between Contrast_N–F_ and Contrast_P–F_ for bilateral precentral gyrus, postcentral gyrus, bilateral OFC, and right MCC. There was a significant difference in neural activation between the two contrasts for both the MCC and OFC. A trend of decreased activation in Contrast_P–F_ (compared to Contrast_N–F_) was observed for the precentral gyrus and postcentral gyrus (*p* < 0.1). *p < 0.001.

For the ROI analysis, the paired *t*-test for the bilateral thalamus revealed that occlusion-induced neural activation (beta values) was comparable between the two contrasts (Contrast_N–F_ vs. Contrast_P–F_, *p* = 0.47). For the primary visual area (V1), the analysis also showed no significant difference between Contrast_N–F_ and Contrast_P–F_ (*p* = 0.45). Figure 2 shows the bar graphs for the Contrast_N–F_ and Contrast_P–F_ in bilateral thalamus as well as the V1 cortices. Similarly, no significant difference was detected between the two contrasts Contrast_N–F_ vs. Contrast_P–F_ for cortical neural activation level in the inferior frontal gyrus (IFG) and supramarginal gyrus (*p* = 0.12 and 0.22, respectively). Regarding the sensorimotor areas, the analysis on the beta values revealed a trend for decreased activation in the positive context, but without statistically significant difference between the two contrasts (*p* = 0.34 and 0.25 for the precentral gyrus and postcentral gyrus, respectively). For the orbitofrontal cortex and MCC, the analysis showed a significant decrease of beta values in the positive compared to the neutral context (ContrastN-F vs. ContrastP-F, *p* < 0.01 and *p* < 0.03 for orbitofrontal cortex and MCC, respectively). Figure 3 showed the bar graphs for the Contrast_N–F_ and Contrast_P–F_ in the sensorimotor areas, OFC and MCC.

## Discussion

The present study examined the brain activation patterns associated with respiratory sensory gating in response to paired inspiratory occlusions while viewing neutral and positive emotional pictures. We hypothesized that the positive emotional stimuli would result in lower brain activation than the neutral emotional stimuli as reflected by BOLD responses. Our results showed that overall, across the three conditions (fixation, neutral, and positive picture series), the positive emotional condition elicited the least neural activation in response to paired inspiratory occlusions. The level of brain activations elicited in the positive emotional condition (relative to fixation) was comparable with the neutral condition (relative to fixation) in several areas including bilateral primary visual cortices, thalamus, the IFG and supramarginal gyrus, whereas bilateral sensorimotor areas showed a trend for decreased neural activation in the positive emotional condition (Contrast_P–F_ compared Contrast_N–F_). In addition, neural activations were significantly lower in the positive emotional condition (Contrast_P–F_ compared Contrast_N–F_) for both the MCC and the OFC.

Based on the activated areas elicited by the paired inspiratory occlusion paradigm in the three conditions, it can be inferred that these brain regions, including the thalamus, hippocampus, sensorimotor cortex, temporal gyrus, frontal gyrus, and cingulate cortex, are involved in mediating respiratory gating functions. This view is consistent with our previous study (Chan et al., 2022a) and is further supported by another study examining voluntary breathing (McKay et al., 2003). Both previous studies demonstrated comparable brain activation patterns than the present study. Interestingly, the Contrast_P–F_ and Contrast_N–F_ showed comparable neural activations in the bilateral thalamus in response to the paired occlusion paradigm in the current study. The important role of the thalamus for neural gating functions was confirmed earlier by an animal lesion study where the thalamic reticular nucleus was crucial in mediating auditory sensory gating function in rats (Krause et al., 2003). However, our present results showed that the bilateral thalamus did not emerge as a brain area, which significantly differentiates between the neutral and positive emotional conditions. This finding suggests the following interpretations: first, the thalamus remains in charge of the sensory level of respiratory neural gating regardless of the emotional context; secondly, emotional context can modulate stimulus-related respiratory sensory inputs to the extent that brain energies or neural resources were allocated into regions other than thalamus.

For bilateral V1 cortex, the effect of increased visual processing was observed during both positive and neutral emotional conditions where the activation pattern of V1 was higher, while being the lowest in the fixation control condition. This confirms that the participants did voluntarily attend to the picture series and processed the stimuli during the experiment. The result also proves that, as can be expected, emotional effects on respiratory sensory gating were not processed by the occipital lobe as no difference between the Contrast_P–F_ and Contrast_N–F_ in the V1 cortices were observed. It has been suggested that emotion-related activity could reflect a general enhancement of visual processing (McAlonan et al., 2008), and perceptual representations in the visual cortex could mirror the content of emotional situations (Miskovic and Anderson, 2018). Therefore, it was anticipated that a comparable activation of V1 during both positive and neutral emotional conditions can be observed.

The present study revealed decreased levels of respiratory gating-induced brain activation under the positive emotional context in the orbitofrontal cortex and the middle cingulate cortex. Two possible mechanisms are proposed: First, the positive emotion context promotes attentional resource re-distribution when processing respiratory sensory information. This notion is supported by von Leupoldt et al. (2010a), where they showed emotional influences on the perception of respiratory sensation by decreased P3 amplitudes of the RREP when watching pleasant and unpleasant emotional picture series compared to neutral picture series (von Leupoldt et al., 2010d). Second, it is possible that the positive emotional context improves sensory gating function. As such, almost all respiratory sensory information is gated, which is supported by a previous RREP study showing a better respiratory sensory gating function in a positive emotional context compared to a neutral emotional context (Chan et al., 2016). Another study found that respiratory sensory gating was decreased in the presence of negative emotional visual stimuli (Chenivesse et al., 2014). In the present study, significantly lower levels of activations in the orbitofrontal cortex and middle cingulate cortex suggest that the need to process emotional aspects of respiratory sensation is much lower in a positive emotional context. However, our results showed that the precentral gyrus was activated in the positive emotional condition, which may be related to sensomotoric aspects related to the stimulus intensity, which means that inspiratory occlusions may involve consciously controlling breathing and putting more effort into breathing during the occluded phase. Nevertheless, the present results still showed a non-significant trend for decreased activation in the positive context for the precentral and postcentral gyrus, which suggests that the effect of occlusion stimuli could still be potentially modulated by emotional context at both sensation and motor action levels. Future research is recommended to test this aspect further by examining the relationships between individuals’ respiratory effort and emotional context during respiratory occlusions.

The middle cingulate cortex and the orbitofrontal cortex were found activated to a lesser extent in the positive emotional context (as shown in Contrast_P–F_ compared to Contrast_N–F_) in response to paired inspiratory occlusions. This finding converges with several previous studies. For example, the cingulate cortex has been identified as a brain area that is directly associated with emotional aspects of dyspnea (Vogt and Laureys, 2005; von Leupoldt et al., 2009a; Herigstad et al., 2017; Stoeckel et al., 2018; Chan et al., 2019). The present finding of the MCC activation is in line with the study of Chan et al. (2019), where the MCC was found to be more activated in high anxious individuals compared to low anxious individuals, while the present study found the MCC less activated in the positive emotional context (Chan et al., 2019). The present result regarding the orbitofrontal cortex converges with several previous studies in which the prefrontal cortex was found activated during dyspneic sensations (Parsons et al., 2001; Evans et al., 2002; Peiffer et al., 2008). Notably, previous studies have more consistently suggested the anterior cingulate cortex (ACC) to be associated with emotional aspects of respiratory perception, whereas fewer studies found the MCC as the neural substrate that was directly associated with these emotional aspects (Chan et al., 2019). The discrepancy may be due to different study designs and methodologies, as the present study used inspiratory occlusion stimuli with the BOLD fMRI technique, whereas most of the previous studies used different stimulus methods including resistive loading, hypercapnia, and mechanical ventilation with PET or fMRI technique. Interestingly, MCC was consistently found activated in previous studies that examined brain substrates induced by hypercapnia, although these studies were not directly testing the effect of affective processing of dyspnea (Banzett et al., 2000; Parsons et al., 2001; Evans et al., 2002), which calls for future neuroimaging studies examining the effects of emotional modulations of hypercapnia-induced respiratory sensations.

Moreover, future studies are required in patients with neurological disorders, who can also suffer from increased respiratory symptoms such as dyspnea (Baille et al., 2019a,b; Das et al., 2019). For example, Baille et al. (2019a) recently demonstrated high levels of dyspnea in patients with Parkinson’s disease, which was related to higher levels of depression and anxiety (Baille et al., 2019a). It may be suspected that these associations are related to disturbed emotional modulations of the neural respiratory sensory gating mechanism, which is further supported by previous findings showing abnormal sensory gating patterns for oropharyngeal stimuli in patients with Parkinson’s disease (Pitts et al., 2016). If found to be true, such disturbances in the interrelationships between emotions and respiratory sensory gating may offer a target for therapeutic interventions in these patients. In addition, our study did not perform resting-state fMRI recordings because the current focus was on brain activities induced by respiratory sensory gating across different emotional contexts. However, functional connectivities between ROIs such as the OFC and MCC may also be examined in future investigations, so that clinical implications in terms of neural bases of emotions and respiratory sensation can be informed for diseases including neurological disorders.

One major limitation in the present study was the lack of independently recorded physiological signals, e.g., participants’ heart beats and respiratory end-tidal PCO_2_, for subsequent noise correction. Although the in-scanner respiration data was recorded by the physiological monitor unit (PMU in PRISMA, Siemens) in this study, the data was not analyzable due to a synchronization failure between fMRI and PMU. Therefore, we adopted the signal extraction method from fMRI data as a remedy for physiological noise correction. Future studies with similar experimental designs are encouraged to include the independently recorded PMU signals during fMRI scanning and to carefully confirm the functionality of PMU with the MRI technical support. In addition, although the present study did not test cognitive processing of respiratory perception, a few studies in the literature supported the notion that emotional context could potentially modulate cognitive awareness of respiratory sensory information processing. For example, Bogaerts et al. (2005) examined the effect of different emotional contexts in modulating respiratory interoceptive accuracy. They discovered that people with negative affective states showed less accuracy in an emotionally distressing context compared to a pleasant context (Bogaerts et al., 2005). In addition, von Leupoldt et al. (2011) tested the impact of unpleasant, neutral and pleasant emotional picture series on the RREP. They showed that the RREP P3 peak (indicative of attentional processing of respiratory sensory stimuli) amplitude was smaller in the unpleasant experimental conditions compared to the neutral condition in lower anxious individuals; however, higher anxious individuals processed more respiratory sensory information cognitively as evidenced by higher RREP P3 peak amplitudes (von Leupoldt et al., 2011). Therefore, future investigations are warranted to examine the role of emotional context in cognitive processing of respiratory interceptive information.

In summary, the present study shows that the emotional context modulates the neural substrates of respiratory sensory gating in a paired inspiratory-occlusion paradigm. Especially a positive emotional context may facilitate a top-down modulation of the respiratory sensory gating function. Imaging results in the present study showed that the MCC and the OFC are the two most prominent brain regions involved in both positive and neutral emotional conditions. Our current results further support the concept that respiratory information processing indeed includes both sensory and affective components and can each inform the future development in clinical rehabilitation strategies. Future studies are recommended to test the emotional impact of clinical interventions on respiratory sensations for managing respiratory symptoms.

## Data availability statement

The raw data supporting the conclusions of this article will be made available by the authors, without undue reservation.

## Ethics statement

The studies involving human participants were reviewed and approved by the Institutional Review Board, Chang Gung Medical Foundation. The patients/participants provided their written informed consent to participate in this study.

## Author contributions

P-YC contributed to the conception and design of the study, organized the database, and wrote the first draft of the manuscript. A-LH and CW performed the statistical analysis. W-PC, C-HC, C-YL, and AvL wrote sections of the manuscript. All authors contributed to the manuscript revision, read, and approved the submitted version.

## Footnotes

1
http://www.neuromorphometrics.com/
